# The future of therapy for alcoholic hepatitis – Beyond corticosteroids

**DOI:** 10.1016/j.jhep.2019.01.016

**Published:** 2019-04

**Authors:** Nikhil Vergis, Stephen R. Atkinson, Mark R. Thursz

**Affiliations:** 1 Department of Hepatology, Imperial College, London, United Kingdom

## Background

Corticosteroids are the only treatment proven to reduce mortality from severe alcoholic hepatitis (SAH), though the benefit is short-lived.^1^ Several potential therapies are currently under evaluation in human clinical trials (Table 1). These therapies target: i) malnutrition; ii) intestinal dysbiosis and its portal translocation; iii) bile acid production; iv) hepatocyte death; v) hepatocyte regeneration; and vi) life-threatening complications of the disease itself.

**Table 1:** **Active published clinical trials for alcoholic hepatitis listed by the U.S. National Library of Medicine at****clinicaltrials.gov****and European Clinical Trials Database at****EudraCT.ema.europa.eu**.

**Pathology**	**Therapeutic target**	**Therapy**	**Trial ID:****clinicaltrials.gov****, EudraCT, PMID**
Portal translocation of gut microbiota	Intestinal dysbiosis	Rifaximin	NCT02116556, EudraCT 2014-002264-33
		Oral vancomycin, gentamycin, meropenem	NCT03157388
		Faecal microbiota transplant	NCT03091010 NCT02458079
		Probiotics *Lactobacillus spp.*	NCT01922895 NCT02335632
	Intestinal mucosal integrity	Zinc Obeticholic acid, Canakinumab, Anakinra	NCT01809132 NCT02039219 NCT03775109
Enterohepatic circulation of bile acids	Farnesoid receptor	Obeticholic acid	NCT02039219
Hepatic inflammation	IL-1β	Anakinra Canakinumab	NCT01809132 NCT03775109
	TLR-4 Non-specific	Anti-LPS IgG Bovine colostrum	NCT01968382 NCT02473341
Hepatocellular injury and repair	Oxidative stress	Metadoxine	NCT02019056 NCT02161653 PMID 24756009
		*N-*acetylcysteine	NCT00863785 PMID 22070475
		S-Adenosyl methionine	NCT00851981 NCT02024295
		Omega 5	NCT03732586
	Hepatocyte regeneration	IL-22	NCT02655510
		G-CSF	NCT01820208 NCT02971306 NCT02442180 NCT01341951 NCT02776059 NCT03703674
Complications	Infection	Co-amoxiclav	NCT02281929
		Ciprofloxacin	NCT02326103
		Rifaximin	NCT02116556
		*N*-acetylcysteine	NCT03069300
	Kidney injury	Terlipressin	EudraCT 2006-002837-19

## Nutritional supplements

Malnutrition is common in this group of patients. Good nutrition is a central tenet of SAH management. Intensive nutrition delivered enterally or parenterally does not appear to confer clinical benefit.^2^ However, achieving a calorific intake >21.5 kcal/kg per day is associated with a reduction in complications and mortality.^2^

## Portal translocation of gut microbiota

Intestinal dysbiosis has been implicated in a range of hepatic diseases. Alcohol consumption causes intestinal dysbiosis and impaired intestinal barrier function. Transfer of intestinal microbiota from humans with SAH to mice confers susceptibility to alcohol-induced steatohepatitis, which can be reversed by faecal microbiota transplantation from humans who drink heavily but do not develop SAH.^3^

Current trials aim to improve bacterial dysbiosis using i) orally administered non-absorbable antibiotics (rifaximin or combined gentamicin, vancomycin and meropenem); ii) probiotics (*Lactobacillus rhamnosus* [NCT01922895] and *acidophilus* [NCT02335632]); or iii) faecal microbiota transplantation.

## Enterohepatic circulation of bile acids

SAH is characterised by marked biochemical and histological cholestasis. The farnesoid receptor (FXR) is a key regulator of bile acid synthesis. Receptor agonism also improves gut barrier function in mouse models of alcohol-related liver disease.^4^ Additional beneficial effects from FXR agonism in ameliorating portal hypertension have been suggested in rodent models of liver disease (reviewed in^5^). Obeticholic acid (OCA) is a semi-synthetic agonist of FXR that has shown promise in non-alcoholic fatty liver disease^6^ and has established efficacy in primary biliary cholangitis.^7^ Clinical trial data are awaited (NCT02039219).

## Immune dysfunction

Immunotherapy for SAH is challenging because hepatic immunopathology exists concurrently with systemic immune defects. Accordingly, attempts to control hepatic immunopathology with systemic immunosuppressants, such as anti-TNFα^8^ or corticosteroid therapy,^9^ are hampered by high rates of infection that offsets clinical benefit. Pre-clinical data suggest that anti-IL-1β therapy does not confer such susceptibility to opportunistic infection and reduces hepatic inflammation, fibrogenesis, stellate cell activation and consequent portal hypertension (NCT02655510, NCT01903798, NCT01809132, EudraCT 2017-003724-79, NCT03775109).

## Hepatocellular injury and repair

Ethanol metabolism and immune responses lead to the generation of reactive oxygen species (ROS) that cause oxidative stress and hepatocellular damage. In single studies, the combination of intravenous *N*-acetylcysteine^10^ or oral metadoxine^11^ with corticosteroids appears to confer a survival benefit and is the subject of ongoing investigation (*N-*acetylcysteine [NCT03069300]; metadoxine [NCT02019056, NCT02161653]) along with S-adenosyl-l-methionine (SAMe) [NCT00851981, NCT02024295]. The efficacy of G-CSF, in part mediated via hepatic regeneration,^12^ has been suggested by small studies^13^ and several trials are in progress aiming to replicate these findings. Similarly, IL-22 has been ascribed hepatoprotective and pro-regenerative features; therapeutic agents are under clinical evaluation (NCT02655510).

## Extrahepatic complications of alcoholic hepatitis

*Infection*: up to 50% of SAH patients will develop infection during the acute illness and nosocomial infections reduce survival.^14^ Defective immune cells have been identified in the systemic circulation of patients with SAH and their presence is associated with the development of infection.[15], [16], [17], [18] Reversing these defects (NCT03069300) or predicting infections are attractive prospects. An alternative approach is to treat all SAH patients with broad-spectrum adjunctive antimicrobial therapy such as co-amoxiclav (NCT02281929) and ciprofloxacin (NCT02326103) and these two agents are currently under evaluation.

*Acute kidney injury*: kidney injury that occurs with SAH portends a poor prognosis. Primed immune cells release a plethora of inflammatory mediators, in particular ROS and nitric oxide, which cause vasodilatation in the splanchnic circulation. The vasopressin analogue terlipressin reduces this vasodilatation and is under investigation for SAH specifically.

## Financial support

We are grateful for support from the Imperial College NIHR Biomedical Research Centre, the Wellcome Trust, UK (294834/Z/16/Z) and the Medical Research Council UK Stratified Medicine Award: Minimising Mortality from Alcoholic Hepatitis (MR/R014019/1).

## Conflicts of interest

MT reports grants and personal fees from Gilead and CN_BIO; personal fees from AbbVie and MSD; grants from Vital Therapeutics. All other authors report no conflict of interest.

Please refer to the accompanying ICMJE disclosure forms for further details.
